# The Use of Regional Anesthesia to Reduce Blood Loss in Isolated Limb Perfusion (ILP)—A Novel Approach

**DOI:** 10.3390/jcm12206542

**Published:** 2023-10-16

**Authors:** Maya Niethard, Heilwig Fischer, Bernhard Gaßmann, Lyubomir Haralambiev, Alexander Tipp, Per-Ulf Tunn

**Affiliations:** 1 Department of Orthopedic Oncology, Sarcoma Center, Helios Klinikum Berlin-Buch, Schwanebecker Chaussee 50, 13125 Berlin, Germany; per-ulf.tunn@helios-gesundheit.de; 2 Center for Orthopedics, Trauma Surgery and Rehabilitation Medicine, University Medicine Greifswald, Ferdinand-Sauerbruch-Straße, 17475 Greifswald, Germany; lyubomir.haralambiev@med.uni-greifswald.de; 3 Department of Oral and Maxillofacial Surgery, Charité–Universitätsmedizin Berlin, Corporate Member of Freie Universität Berlin, Humboldt-Universität zu Berlin and Berlin Institute of Health, Augustenburger Platz 1, 13353 Berlin, Germany; heilwig.fischer@charite.de; 4 Center for Musculoskeletal Surgery, Charité–Universitätsmedizin Berlin, Corporate Member of Freie Universität Berlin, Humboldt-Universität zu Berlin and Berlin Institute of Health, Augustenburger Platz 1, 13353 Berlin, Germany; 5 BIH Charité Clinician Scientist Program, Charité–Universitätsmedizin Berlin and Berlin Institute of Health, BIH Biomedical Innovation Academy, Charitéplatz 1, 10117 Berlin, Germany; 6 Meso International GmbH, Markt 21, 09648 Mittweida, Germany; bg@sonowin.de; 7 Department of Anesthesiology, Helios Klinikum Berlin-Buch, Schwanebecker Chaussee 50, 13125 Berlin, Germany; alexander.tipp@helios-gesundheit.de

**Keywords:** isolated limb perfusion, blood loss, sarcoma, regional anesthesia, extracorporeal circulation, hyperthermia

## Abstract

Background: Isolated limb perfusion (ILP) for soft tissue sarcomas (STS) is usually performed with tumor necrosis factor alpha (TNF-α) and melphalan. ILP regularly leads to a total blood loss (BLt) of 1.5–2 L/patient. Blood inflow from the central blood circulation to the limb is influenced by unstable pressure gradients and pain reactions after the administration of melphalan. With perioperative regional anesthesia (RA), pain levels can be reduced, and the pressure gradient stabilized resulting in a reduced BLt. The aim of this study was to compare the BLt with and without RA in patients with ILP during circulation of drugs. Methods: Patients were treated according to the following protocol: After the establishment of limb circulation, ILP was started with the administration of TNF-α. Half the dose of melphalan was given as a bolus after 30 min, and the remaining dose was continuously administered in the following 30 min. The extremity was washed out after 90 min. ILP with perioperative RA (supraclavicular plexus block/peridural catheter) was performed prospectively in 17 patients and compared to a matched retrospective control group of 17 patients without RA. BLt was documented and perioperative anesthesiological data were analyzed for response rates after the application of melphalan (RaM). Results: BLt and RaM tended to be lower for the intervention group with RA if compared to the control group without RA in all analyses. The trend of lower BLt and RaM in ILP with RA was more pronounced for the upper extremity compared to the lower extremity. Results were not statistically significant. Conclusion: These findings indicate that the use of RA can help to stabilize hemodynamic anesthetic management and reduce the BLt in ILP, especially during perfusion of the upper extremities.

## 1. Introduction

Isolated limb perfusion (ILP) is an established surgical therapeutic modality in the treatment of soft tissue sarcomas. In 2020, it was anchored in Germany in the S3 guideline on adult soft tissue sarcomas, register number 032-044OL [1]. The procedure was first described in 1952 and adapted by Eggermont in 1992 for the treatment of soft tissue sarcomas using melphalan and TNF-a [2]. It is used as a local high-dose chemotherapy combined with hyperthermia to improve the resectability of the tumor and prevent imminent limb amputation in patients with locally advanced tumors. Overall response rates of 68% and overall limb salvage rates between 73.8% and 88% can thus be achieved [3,4,5].

The incidence of soft tissue sarcoma is low and high-grade tumors are often treated with systemic chemotherapy [6]. The indication for neoadjuvant ILP alone is rare, which is why it is often indicated interdisciplinary, as an additional neoadjuvant treatment or with palliative intention. Therefore, the oncological outcome is not the focus of this study. Due to the complexity of the isolated hyperthermic extremity perfusion with high logistical effort, the intervention is only performed in a few sarcoma centers. Statistically relevant case numbers can only be achieved in an international multicentric setting [3], which, however, entails different procedures in the perioperative implementation regarding anesthetic management and surgical technique. For the evaluation of an add-on to the surgical procedure, only an in-house comparison seems reliable where the performing team for the procedure (surgeons/anesthesia/nuclear medicine/medical physics) can be assumed to be a constant parameter.

By decoupling the extremity circulation from the body circulation, two separate compartments are created, whereby the chemotherapeutics are only applied to the extremity. Leakage control is performed by indium- and technetium-labeled erythrocytes with appropriate nuclear medicine probes over the body and extremity circulation, respectively. A maximum leakage rate of 5% for TNF-α and 7% for melphalan from the extremity circulation into the systemic circulation can be tolerated during the entire perfusion period of 90 min [5,7,8]. Anesthesiological management includes maintaining a stable pressure gradient between the circulatory compartments during perfusion to minimize inflow rates in both directions. By using the extremity tourniquet, the flow through peripheral vessels is eliminated or reduced to a minimum. During the perfusion, melphalan concentrations that are about 10–15 times higher than in systemic chemotherapy are given locally [9].

Due to these complex processes, ILP regularly leads to a blood loss of approx. 1000–1500 mL/patient, which results from the blood volume for filling the heart-lung machine (450 mL), the discarded cytostatic-contaminated blood volume of the extremity, and the blood volume as inflow from the systemic circulation into the limb circulation during perfusion. While the filling volume of the heart-lung machine and the washed-out blood volume of the extremity cannot be influenced, observations of >200 ILPs showed that, especially after administration of the melphalan bolus, there is a sudden inflow from the systemic circulation into the circulation of the extremities, while at the same time, hemodynamic instability occurs in anesthesia management. Both factors lead to an increase in blood volume, which must be discarded after the completion of ILP and appears to be directly related to the administration of melphalan. Melphalan is a cytostatic drug from the group of alkylating agents, which is used in the treatment of various types of cancer, e.g., multiple myeloma, and in the preparation for hematopoietic stem cell transplantation. The effects of melphalan are based on the cross-linking of DNA, which leads to the inhibition of DNA replication. For a high-dose therapy, administration is parenteral [10]. The most common possible adverse effects include nausea, vomiting, diarrhea, mouth sores, hair loss, bone marrow suppression, muscle atrophy, muscle fibrosis, muscle pain, and warmth or burning at the injection site [11]. The product information sheet for EVOMELA^®^ (melphalan) lists the following under the point of overdose: “Overdoses, including doses up to 290 mg/m^2^, have produced the following symptoms: severe nausea and vomiting, decreased consciousness, convulsions, muscular paralysis, and cholinomimetic effects.” [12]. ILP is performed with melphalan dosages that are 10–15 times higher than systemic therapy, so that an accumulation of the side effects can be assumed [9,12]. The intensification of the cholinomimetic effects would thus lead to a local parasympathetic reaction, followed by a sudden increase in blood flow from the systemic circulation into the extremity. Since the extremity tourniquet minimizes or, in the best case, excludes the passage of blood through the soft tissue compartment, it can be assumed that the blood passage must take place via the bone. The amount of blood that has passed over can be read immediately and quantitatively from the reservoir of the extracorporeal circulation (ECC) machine during the perfusion.

**Figure 1:**
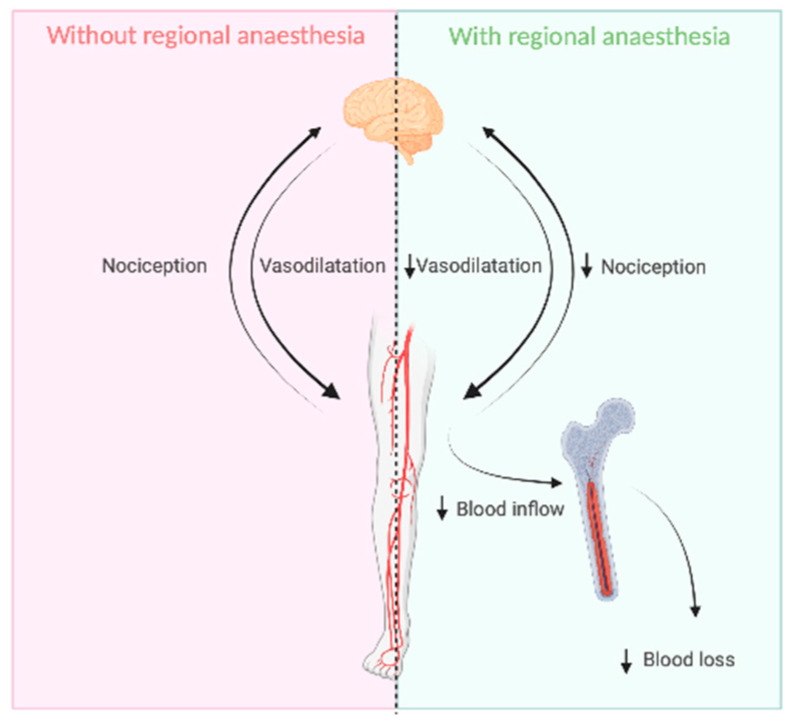
Targets of RA to reduce blood inflow into the extremity through the bone after the application of melphalan (created online with biorender.com on 2 February 2023).

ILP is a technically challenging procedure and requires a combined effort including the surgical and anesthesiological team, medical physicists, and nuclear medicine specialists. Hemodynamic and metabolic monitoring is necessary, and it is a demanding task to manage significant hemodynamic and metabolic changes due to fluid shifts and cytokines release [8,13]. Due to these complex processes, ILP regularly leads to a blood loss of approx. 1000–1500 mL/patient [14,15]. With >200 ILPs in the last 15 years, we observed a constant low-threshold systemic blood loss from the central blood circulation to the limb circulation with the start of ILP. We assume that unstable pressure gradients on both sides of the tourniquet and a local reaction after the administration of melphalan lead to an unavoidable blood inflow into the limb across the tourniquet and via the bone [16]. A case of a verified leakage during ILP through bone marrow by venography has been described by Katsarelias et al. in 2018 [17] (Figure 1).

Several efforts have been made to optimize the ILP technique. Trying to reduce the side effects of ILP on the patients, Grunhagen and Deroose et al. showed that reducing the TNF-a dose resulted in a decrease in tissue-damaging side effects and serious soft tissue complications, including amputations, without losing the therapeutic effect [18,19]. Hohenberger et al. investigated whether the administration of pentoxifylline can reduce the occurrence of a systemic inflammatory response syndrome (SIRS) to reduce systemic side effects after the procedure. However, the approach was not pursued further [20].

Anesthetic efforts during ILP focus on sustaining normovolemia and normotension to ensure adequate systemic organ perfusion whilst simultaneously limiting blood loss by maintaining a stable pressure gradient between systemic and limb circulation. Therefore, circulatory parameters including heart rate and invasive blood pressure as well as indicators of fluid responsiveness such as pulse pressure variation should be assessed to determine fluid needs and vasopressor requirements. Elevated blood pressure, posing a potential bleeding risk resulting from an increased pressure gradient, should be treated via optimization of pain management and deepening of anesthesia [13,14].

There has been only limited research on the use of perioperative measures to reduce blood loss in ILP up to this day. Patients with soft tissue sarcomas and an indication for ILP often present in a reduced general condition during their oncological treatment after previous therapies such as systemic chemotherapies or radiation therapy. It is, therefore, of enormous benefit to keep the blood loss in these patients as low as possible. A shorter hospital stay, including the rehabilitation phase, gives oncological patients more time at home and shortens the time it takes to resume necessary follow-up therapies. Avoiding blood transfusions reduces the increased risk of transfusion-associated complications in oncology patients [21] and conserves healthcare system resources.

Melphalan has been shown to induce a proinflammatory cytokine/chemokine milieu, resulting in spike levels of cytokines including IFN-γ, IL-22, IL-10, IL-5, IL-18, and IL-27, and chemokines including CCL2, CCL7, CXCL1, and CXCL10 [21]. During the clinical course of ILP, the administration of the melphalan bolus results in a hemodynamic reaction in the patient with a measurable increase in blood volume in the reservoir, necessitating an adjustment of the anesthetic management. We suspect that the strong local tissue irritation at non-physiological dosages triggers, among other things, an increased pain stimulus in the extremity directly after the administration of melphalan. Perioperative regional anesthesia (RA) decreases the local pain stimulus by temporarily blocking the nerves supplying the extremities with local anesthesia, and is routinely used in surgical procedures of the extremities [22] (Figure 1).

Since both ILP and RA are established procedures, the combination of the two is not expected to increase the risk to patients. The aim of this study was to investigate whether the suppression of the local pain sensation in the extremity by perioperative RA can reduce measurable effects directly after the administration of melphalan during ILP, and minimize the hemodynamic changes and fluid shifts, leading to a reduction in blood flow from the systemic circulation to the extremity circulation and resulting in a reduced blood loss during perfusion time with circulating chemotherapeutic agents (Figure 1).

## 2. Materials and Methods

### 2.1. Study Design

This study was approved by the Ethics Committee of the Berlin Medical Association under number Eth-63/22. This single-center study reviews all patients who underwent a multimodal treatment protocol at the Sarcoma Center Berlin-Buch due to an advanced soft tissue sarcoma between 2016 and 2021. Inclusion criteria were high-grade soft tissue sarcoma of the extremities, scheduled ILP with a consented indication for the procedure in the multidisciplinary sarcoma tumor board, and written informed consent. ILP was carried out by the same team of surgeons and anesthesiologists on all patients according to a standard operating procedure. For the prospective intervention group, 17 patients received their ILP in combination with RA between 11/19 and 12/21. Written informed consent for the treatment with either a peridural catheter or interscalene block for RA was obtained separately from each patient.

The retrospective control group consisted of 17 consecutive patients who received their treatment prior to the intervention group, i.e., before 11/19. Patients were matched according to sex, upper and lower extremity localization, and dosage of TNF-a. Three children aged below 16 years (7, 13, and 15 years) in the prospective intervention group with an ILP of the lower extremity were referred to the upper extremity group because of more comparable extremity volume and identical dosage of TNF-a.

The parameters collected were gender, age, neoadjuvant oncological therapy, preoperative levels of hemoglobin (Hb) and hematocrit (Hc), postoperative levels of Hb and Hc (day 1), surgical access level, volume of the extremity, dosage of TNF-α, dosage of melphalan, duration of surgical procedure, duration of circulation of TNF-α + melphalan, nuclear medicine leak rate, total blood loss, blood loss after application of melphalan for the intervention group, hemodynamic response after application of melphalan (RaM), transfusion of red blood cells (RBC), and substituted volume during ILP.

The exclusion criteria were incomplete course of ILP due to a leak rate >10% and perioperative complications with the RA device.

The primary endpoints were BLt and RaM during ILP.

### 2.2. Surgical Treatment

All patients were operated on under general anesthesia with endotracheal intubation. Lower extremity ILP was performed via an iliacal, femoral, or adductor approach with dissection and cannulation of the corresponding artery and vein. An axillary and brachial approach was used for the upper extremity. The remaining collateral vessels were compressed using an Esmarch rubber band and the cannulas were then connected to the extracorporeal circulation (ECC). The perfusion was performed with a pump flow rate between 100– and 800 mL/min to ensure a stable level in the reservoir. The total perfusion time after the application of drugs was 90 min in all patients. The perfusion started with the administration of TNF-a as a bolus followed by a 50% melphalan dosage as a bolus after 30 min, and the administration of the remaining 50% melphalan continuously for another 30 min. The highest perfused tissue temperature was kept at a maximum level of 39.8 °C (mild hyperthermia). The dose of TNF-a was 1 mg for the arm and children’s legs and 2 mg for the leg in adults. The dose of melphalan was calculated as 10 mg/L of perfused tissues for the lower limb and 13 mg/L for the upper limb. At the end of the perfusion, the limb was irrigated with 3000–4000 mL of Ringer’s acetate until a clear rinse became visible in the ECC system (Fresenius Kabi AB, Uppsala, Sweden) [11].

### 2.3. Anesthetic Care and Monitoring

For preoperative preparation, blood was drawn for complete blood count, electrolytes, and coagulation studies. A minimum of two units of RBC were ready for each patient. Anesthesia was induced under standard monitoring using propofol 2 mg/kg body weight (BW), sufentanil 0.3 to 0.5 mcg/kg BW, and atracurium 0.5 mg/kg BW for neuromuscular blockade. After orotracheal intubation, an arterial line, a central venous line, and a urinary catheter were placed. Balanced crystalloid solutions (Jonosteril Fresenius Kabi, Bad Homburg, Germany) were administered as needed. Anesthesia was maintained with either desflurane or sevoflurane using minimal flow (Aisys, Datex Ohmeda, Waukesha, WI, USA), or by propofol infusion (Agilia Injectomat, Fresenius Vial S.A.S, Brézins, France). FiO_2_ was set between 0.4 and 0.6.

In patients with upper limb ILP, a single-shot supraclavicular brachial plexus block with prilocaine 1% 15 to 20 mL was performed preoperatively under ultrasound guidance through in-plane puncture in the short axis view. For this particular technique, the dosage of local anesthetic was referred to by the recommended literature ranging from 10 to 30 mL [22,23,24,25]. In order to ensure a sufficient effect and standardize the procedure, the dosage in our study protocol was set to 15–20 mL, giving the anesthesiologist an individual range for decision based on the distribution of the local anesthetics in the ultrasound image. In lower extremity ILP, a single dose of 15 to 20 mL prilocaine 2% was given prior to isolated circulation via a lumbar epidural catheter inserted on the day before surgery. For the three children aged < 16 years included in the intervention group, the dosage of local anesthetics administered via the epidural catheter was adjusted in relation to their BW.

**Figure 2:**
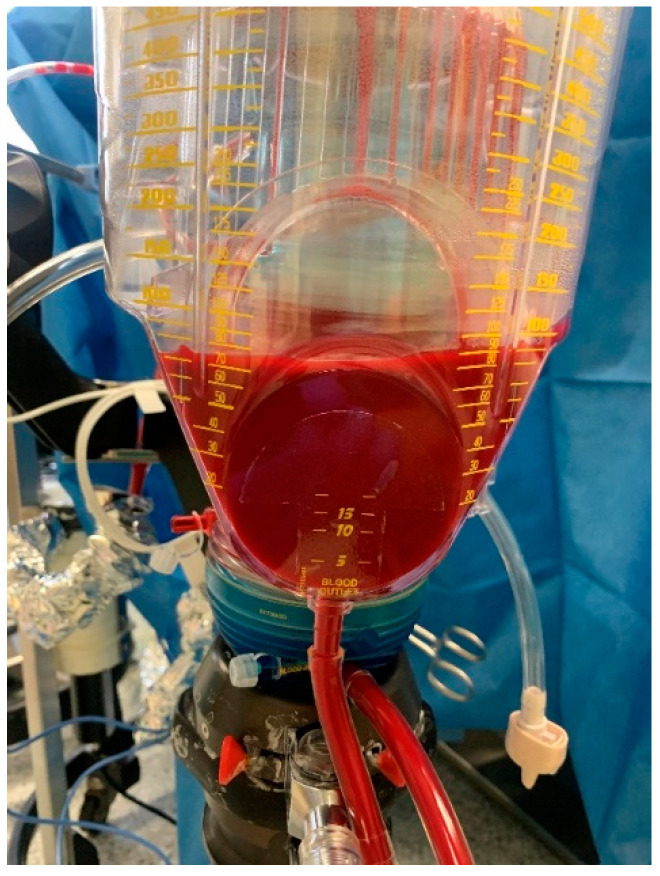
ECC reservoir.

In order not to overlook a postoperative compartment syndrome, the epidural catheter was not used for further pain therapy; instead, sufentanil was given at infusion rates of 0.1 to 0.2 mcg/kg BW per hour during the perfusion. The peridural catheter was removed on the first day after surgery. Postoperative pain management was achieved by oral administration of metamizol 4 g/day and oxycodon 20 mg/day, and was reduced as soon as pain relief was achieved. To maintain systemic perfusion pressure and to prevent the shunting of blood between the systemic circuit and isolated limb, norepinephrine was administered continuously at infusion rates of 0.05 to 0.15 mcg/kg BW per hour via the central venous line. To enable extracorporeal circulation, 200 IU/kg BW heparin was injected 3 min before the canulation of the vessels. During the 90 min perfusion time, blood loss was monitored by the perfusionist on the metric scale of the ECC reservoir (Figure 2), and data were collected in an Excel sheet every 5 min.

For the data collected since 2016, the same perfusionist routinely documented BLt during ILP by reading out the ECC reservoir. The values can be read in 50 mL increments on the upper scale. The calculation of BLt starts after the safe cannulation of the extremity vessels and ends with the completion of the rinsing of the extremity. It is composed of a constant of 450 mL of blood required to fill the ECC, the blood volume of the limb, and the amount of blood that leaks to the limb during the procedure. The blood volume of the limb is removed by washing out after the perfusion time has elapsed. Rinsing is continued until less than 10% of the activity of the labeled erythrocytes is detectable. The BLt volume is read directly on the ECC reservoir, and the amount of rinsing fluid is subtracted. Blood loss from surgical bleeding during dissection prior to the start of circulating drugs or during the postoperative clinical course is excluded.

Hb and Hc levels were collected on the admission day prior to surgery and on the morning of the first postoperative day in the intermediate care unit.

Elevation or drop of blood pressure of more than 20% [mm Hg], a change in heart rate by more than 20% [beats/minute], an increased need of noradrenalin of more than 50%, or an increased need of cristalloid fluids of more than 50% were considered response criteria for RaM.

Blood transfusions were not given during the isolated limb perfusion due to possible interference with the radiolabeled autologous erythrocytes used for shunt monitoring.

The patients were safely extubated and transferred to our intermediate care unit for continued monitoring and continuous infusion therapy after ILP. To prevent acute kidney injury, high-volume forced diuresis was induced by continuous crystalloid infusion at a rate of 200 mL per hour, and 10 mg furosemide i.v. was administered if fluid intake was greater than outtake in a period of two hours. Blood was drawn daily for creatine kinase and myoglobin as diagnostic parameters of rhabdomyolysis. In the operated limb, pulse as well as sensory-motor signaling were surveyed every two hours on the surgery day and every four hours on the following days.

### 2.4. Statistical Analysis

Data were collected in an Excel sheet (Microsoft^®^ Excel^®^ 2016 (16.0.5395.1000)) and analyzed with SAS^®^ 9.4. Graphs were created with STATA/IC 11.0 for Windows (32-bit), StataCorp LP 2009.

To compare the two groups regarding BLt, the Shapiro–Wilk test was used to check whether the data were normally distributed. Since the assumption of normal distribution was rejected (*p*-value ≥ 0.1), the Mann–Whitney U test was used. To compare the frequency distributions of the response rates of the two groups, the chi-square test was used. If more than 20% of the cells in the table had an expected frequency of less than five, Fisher’s exact test was used instead. *p*-values of < 0.05 were deemed statistically significant.

## 3. Results

### 3.1. Patients

Between 11/2019 and 12/2021, 19 patients received a RA for their ILP and were screened for the intervention group. Two patients had to be excluded because of a dislocation of the peridural catheter during the intervention and due to the early termination of ILP due to intolerable leakage > 10%, leaving seventeen patients for analysis in the intervention group. For the control group, patient records of patients with ILP without RA were retrospectively reviewed from 11/2019 to 01/2016. A total of 35 patients were screened until the control group matched 17 patients for sex, localization (upper/lower extremity), and TNF-a dose.

The median age of the 34 patients analyzed was 65 years (range 7–83 years). *N* = 10 were females. Both groups were comparable in terms of the surgical approach, the volume of the perfused extremity, the dosage of melphalan, the duration of the surgical procedure, and the duration of drug circulation (TNF-a and melphalan). Within the groups, the upper extremity was treated in eight patients (47.1% of seventeen) and the lower extremity in nine patients (52.9% of seventeen). Approaches were axillary (*n* = 1), brachial (*n* = 12), iliacal (*n* = 8), femoral (*n* = 12), and adductor (*n* = 1). Patient demographics and characteristics are depicted in Table 1. Supplementary Table S1 shows the detailed parameters for each patient. In the intervention group, three children aged 7, 13, and 15 years received an ILP of their leg with a femoral approach but were analyzed as ILP of the upper extremity because the extremity volume was more comparable to the volume of an adult’s arm, resulting in a reduced dosage of melphalan; drug therapy was also adapted to ILP of the upper extremity with a reduced dosage of 1 mg TNF-a. The dosage of local anesthetic was adapted to their BW. The outcome variables BLt and RaM were analyzed in the total collective or as a subgroup analysis with regard to upper and lower extremities.

### 3.2. Total Collective (n = 34)

**Figure 3:**
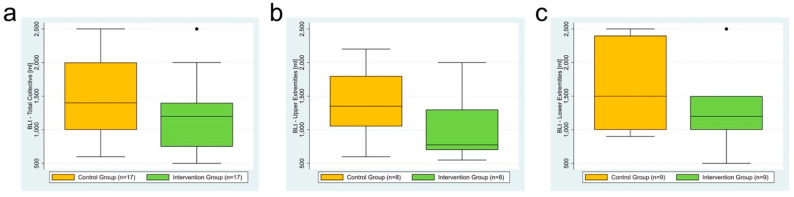
(**a**–**c**) Boxplot of BLt in the intervention group vs. control group: (**a**) total collective, (**b**) subgroup analysis: upper extremity, and (**c**) subgroup analysis: lower extremity.

BLt in the intervention group was compared to BLt in the control group. With a median of 1200 mL, BLt tended to be lower in the intervention group than in the control group with a median of 1400 mL (*p* = 0.124) (Table 2a, Figure 3a).

**Figure 4:**
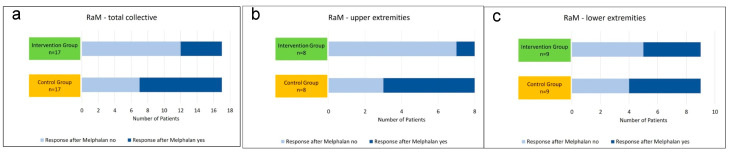
(**a**–**c**) RaM in the intervention group vs. control group: (**a**) total collective, (**b**) subgroup analysis: upper extremity, (**c**) subgroup analysis: lower extremity.

RaM in the intervention group was compared to RaM in the control group. With a response rate of 29.4%, RaM was lower in the intervention group than in the control group with a response rate of 70.6% (*p* = 0.084) (Table 3a, Figure 4a).

### 3.3. Subgroup Analysis: Upper Extremity (n = 16)

For subgroup analysis of the upper extremity group, BLt in the intervention group was compared to BLt in the control group. With a median of 775 mL, BLt tended to be lower in the intervention group than in the control group with a median of 1350 mL (*p* = 0.155) (Table 2b, Figure 3b).

RaM in the intervention group was compared to RaM in the control group. With a response rate of 12.5%, RaM was lower in the intervention group than in the control group with a response rate of 87.5% (*p* = 0.119) (Table 3b, Figure 4b).

### 3.4. Subgroup Analysis: Lower Extremity (n = 18)

For subgroup analysis of the lower extremity group, BLt in the intervention group was compared to BLt in the control group. With a median of 1200 mL, BLt tended to be lower in the intervention group than in the control group with a median of 1500 mL but the Mann–Whitney U test did not confirm a difference (*p* = 0.592) (Table 2c, Figure 3c).

RaM in the intervention group was compared to RaM in the control group. With a response rate of 44.4%, RaM was lower in the intervention group than in the control group with a response rate of 55.6%, but the related *p* value for group comparison equaled 1 (Table 3c, Figure 4c).

## 4. Discussion

The aim of this study was to investigate whether the suppression of the local pain sensation in the extremity by perioperative RA can reduce hemodynamic changes and fluid shifts directly after the administration of melphalan during ILP. The hypothesis was that perioperative RA would lead to a reduction in blood flow from the systemic circulation to the extremity circulation, resulting in a reduced blood loss during perfusion time with circulating chemotherapeutic agents. We found a trend for a reduced RaM and BLt for the intervention group with RA compared to the control group without RA. Trends were more pronounced in the subgroup analysis of the upper extremity, but results were not statistically significant.

The anesthetic management of patients undergoing ILP requires special attention and severe hemodynamic changes can be seen during administration of drugs [14]. Ruschulte et al. [13] retrospectively reviewed 17 cases, regarding the anesthetic and hemodynamic changes during ILP for malignant melanoma with melphalan only, and discussed their clinical practice of preoperative, perioperative, and intraoperative patient care. They observed a decreased adjusted shock index (heart rate/systolic blood pressure) as a measurable response to circulating melphalan during the course of ILP. Three patients experienced transient hypotension and one patient went into cardiac arrest at the time of extubation, demonstrating that the administration of melphalan can lead to intensive hemodynamic changes. But they could not observe significant changes in hemodynamics. Since we observed that hemodynamic changes requiring compensation in anesthetic management occurred more often directly after the application of melphalan, we focused on RaM and saw a lower response rate of 29.4% in the intervention group than in the control group with a response rate of 70.6% (*p* = 0.084). Although not statistically significant, the trend gives a hint that there might be mechanisms to influence RaM. While in the upper extremity five out of eight patients in the control group showed a hemodynamic response after melphalan, only one out of eight in the intervention group did.

It is well known that high leakage rates during ILP have a direct impact on hemodynamic changes mostly caused by a TNF-a-mediated toxicity [8,26]. Christoforidis et al. [27] compared the hemodynamic response in ILP in a TNF group versus a non-TNF group. Significant changes were observed in the TNF group (*p* < 0.006) regarding the mean arterial pressure, decrease in the systemic vascular resistance index, and an increase in temperature, heart rate, and cardiac index. These hemodynamic alterations started when the ILP tourniquet was released. Our intervention group for the lower extremity differs from the other groups with a median leakage rate of 4% and an outlier of 10%, while the other three comparison groups each had a leakage rate of <1%. This could be the reason why no difference in RaM was found in the lower extremity, with 4/9 patients in the intervention group and 5/9 in the control group showing a response. But a high leak rate was not necessarily related to RaM.

Beasley et al. [28] reported on the pharmacokinetics of melphalan in hypothermic isolated limb perfusion optimizing the exposure of tissues to melphalan while minimizing the peak perfusate concentration of melphalan in order to minimize toxicity during perfusion. Toxicity after ILP with melphalan has been shown to be related to the peak concentration of melphalan in the perfusate [29], which correlates with our perception of hemodynamic changes immediately after melphalan administration where our hypothesis is that one of the triggers might be a local pain stimulus. Nieweg et al. [30] described their technical experience for ILP at a single center, and assessed epidural anesthesia as risky in a patient who is fully heparinized. They did not recommend the use of RA during ILP because of generally assumed side effects like induction of vasodilatation and predisposition of leakage of blood from the systemic circulation to the perfusion circuit, but they did not present any data. To avoid complications after heparinization we decided to establish peridural catheters for lower leg perfusion on the day before surgery. A single shot suprascalene block was used in the upper extremity.

To our knowledge, the use of regional anesthesia during ILP has not been studied otherwise. However, a reduction in intraoperative blood loss has been shown to be associated with a better postoperative outcome for patients after arthroplasty. Perioperative measures such as the use of tranexamic acid, the use of an extremity tourniquet, and intracapsular injected substance mixtures aim for a reduction in blood loss during arthroplasty and can show positive effects regarding reduced transfusion rates, faster rehabilitation phase, and shorter length of hospital stay [31,32,33,34,35,36]. Goubran et al. reported that the administration of RBCs from donors has an immunomodulatory effect and might have a negative outcome on ILP response rates [21], which would be counterproductive for our cohort, strengthening the need to avoid perioperative blood transfusions. Another interesting option to reduce blood loss during ILP was reported by Corderfeldt et al. They replaced an erythrocyte-based prime solution with a crystalloid-based prime solution while maintaining the regional metabolic oxygen demand during ILP, and showed no significant improvement with the addition of packed red blood cells into the prime solution. They recommended that a crystalloid-based prime solution should be used [37]. If used in our cohort, the use of a crystalloid priming solution would save 450 mL of blood per patient, as in our study setup the ECC was filled with 450 mL autologous blood for each patient. The two approaches can complement each other, as the use of RA aims to influence blood loss through a different mechanism.

The selection of measurable parameters to obtain a reliable statement on blood loss during ILP leads to several challenges. Calculation methods including Hb and Hc levels as used for the calculation of blood loss in joint replacement surgery [38,39,40,41,42] are difficult to apply to oncologic patients undergoing ILP. Peng et al. used total blood loss, Hb and Hc drift, and the need for a blood transfusion as primary outcome measures in a randomized controlled study to evaluate postoperative blood loss in total knee arthroplasty [41]. Back in 1962, Nadler et al. reported an arithmetic formula to calculate the patient’s blood volume [43], which serves as a template for Gross et al. who developed a calculation method for allowed blood loss during surgery, corrected for dilution [42]. They stated that their logarithmic formula assumes a relatively slow and steady blood loss with maintenance of intravascular volume with erythrocyte-free solutions. Neither of these conditions are met during ILP. Namendys-Silva et al. [15] reported on the clinical characteristics of critically ill cancer patients undergoing ILP with TNF-a and melphalan. Parameters such as diagnosis, localization, duration of surgery, and perioperative volume replacement were comparable to our cohort. The incidence of organ dysfunction according to the Mexican Sequential Organ Failure Assessment (MEXSOFA) was 90.5%, and 54% of patients required red blood cell transfusion, reflecting the high transfusion requirements of cancer patients. Fifteen out of our thirty-four patients had undergone oncological pretreatment such as chemotherapy and/or radiotherapy in the previous 6 months. Three patients presented with an Hb of < 10 g/L on admission, all requiring postoperative RBC transfusion, and one of them receiving an additional RBC preoperatively. But neoadjuvant therapy was not always associated with postoperative transfusion. Postoperative Hc levels showed extensive dilution which can be attributed to the intraoperative high-volume requirement caused by a SIRS-reaction and the postoperative forced diuresis with an infusion volume of 200 mL/h for at least 24 h. Hc even dropped by up to 50% in some of our cases. As another limitation, the measurement of Hb and Hc will include blood loss due to surgical bleeding from dissection and postoperative bleeding into the wound drainage. Pre- and postoperative Hb and Hc levels were, therefore, not considered reliable parameters for the estimation of blood loss during ILP. Standardized setup for ILP at our institution allowed a reproduceable and reliable way to document the total blood loss during circulation of drugs in ILP by reading values directly from a metric scale from the reservoir of the closed ECC unit.

The intervention group with regional anesthesia showed a trend for lower blood loss during ILP and a lower rate of hemodynamic responses after the administration of melphalan bolus, supporting our hypothesis that RA might have a positive effect on handling side effects regarding nociception and cholinomimetic effects. Both factors are more pronounced in the subgroup analysis for the upper extremity than for the lower extremity. One possible explanation could be a reduced proportion of soft tissue in the upper extremity, and thus a better compression of the vessels by the tourniquet, as well as a higher extremity volume resulting in a greater extravasal distribution space in the lower extremities.

Our study does have major limitations with small case numbers from a single center which are due to the low incidence of sarcomas with indication for ILP. We used a retrospective cohort as the control group, and the patient population is inhomogeneous in terms of age and previous oncological therapies. Therefore, the results in this study must be analyzed with extreme caution.

But this study also strengthens the importance of anesthetic management during ILP focusing on hemodynamic changes after the administration of TNF-a and melphalan, and perioperative RA may be a useful tool to optimize patient management during ILP. Especially in the field of oncology, everything should be done to avoid unnecessary blood loss and blood transfusions.

## 5. Conclusions

The results of this pilot study analyze for the first time the use of RA and its impact on blood loss and hemodynamic response rates during the administration of TNF-a and melphalan in ILP. Although not statistically significant, there was a trend for a reduced BLt and RaM for ILP combined with RA compared to the control group without RA. Trends were more pronounced in the subgroup analysis of the upper extremity. ILP remains a very effective but very challenging surgical procedure. As a standard procedure in perioperative anesthesiological management, RA can easily be implemented as an add-on in surgical routine. Perioperative RA as an additional measure during ILP should be further investigated and may be beneficial for selected patients, especially in an oncological setting.

## Figures and Tables

**Table 1:** Patient demographics and characteristics; metric variables are described by median and extrema.

	Control Group		Intervention Group	
Variable	Upper Extremity	Lower Extremity	Upper Extremity	Lower Extremity
***N***	8	9	8	9
**Sex (m:f)**	7:1	5:4	7:1	5:4
**Age**	60.5 yrs (17–83)	51 yrs (23–81)	51.5 yrs (7–82)	77 yrs (49–83)
**Surgical approach**	brachial *n* = 7 axillary *n* = 1	iliacal *n* = 4 adductor *n* = 1 femoral *n* = 4	brachial *n* = 5 femoral (child) *n* = 3	iliacal *n* = 3 femoral *n* = 6
**Extremity volume**	3.22 L (1.89–4.96)	10.95 L (7.85–14.37)	3.69 L (2.4–7.06)	10.3 L (7.83–17.64)
**Dosage TNF-a**	1 mg	2 mg	1 mg	2 mg
**Melphalan dosage**	30 mg (20–50)	100 mg (80–120)	35 mg (25–60)	80 mg (60–120)
**Duration of surgical procedure**	238 min (201–286)	237 min (211–260)	227 min (132–247)	228 min (190–311)
**Circulation of TNF-a + Melphalan**	90 min (88–97)	92 min (86–94)	94 min (69–95)	93 min (73–97)
**Leak rate**	1.5% (1–5)	1% (0.5–8)	1% (1–5)	4% (1–10)
**Total blood loss (BLt)**	1350 mL (600–2200)	1500 mL (900–2500)	775 mL (550–2000)	1200 mL (500–2500)
**Blood loss after melphalan (BLm)**	-	-	0 mL (*n* = 3) 150 mL (70–250) (*n* = 5)	0 mL (*n* = 6) 300 mL (100–400) (*n* = 3)
**Response after melphalan (RaM)**	Yes = 5 No = 3	Yes = 5 No = 4	Yes = 1 No = 7	Yes = 4 No = 5
**Transfusion of RBCs**	1 RBC (*n* = 0) 2 RBC (*n* = 1)	1 RBC (*n* = 0) 2 RBC (*n* = 2) 4 RBC (*n* = 1)	1 RBC (*n* = 4) 2 RBC (*n* = 1)	1 RBC (*n* = 2) 2 RBC (*n* = 1)
**Intraoperative volume substitution**	5500 mL (3000–7000)	5000 mL (4000–9000)	4500 mL (3000–7000)	5000 mL (3000–8000)

**Table 2:** (**a**–**c**) BLt depending on the study group.

Variable	Study Group	N	Min	Q1	Median	Q3	Max
(**a**) *total collective*							
BLt [mL]	control	17	600	1000	1400	2000	2500
	intervention	17	500	750	1200	1400	2500
(**b**) *subgroup analysis: upper extremity*							
BLt [mL]	control	8	600	1050	1350	1800	2200
	intervention	8	550	700	775	1300	2000
(**c**) *subgroup analysis: lower extremity*							
BLt [mL]	control	9	900	1000	1500	2400	2500
	intervention	9	500	1000	1200	1500	2500

min = minimum, Q1 = lower quartile, Q3 = upper quartile, max = maximum.

**Table 3:** (**a**–**c**) RaM depending on the study group.

		(a) *Total Collective*				(b) *Subgroup Upper Extremity*				(c) *Subgroup Lower Extremity*			
		Control *n* = 17		Intervention *n* = 17		Control *n* = 8		Intervention *n* = 8		Control *n* = 9		Intervention *n* = 9	
Variable	Value	*n*	% *	*n*	% *	*n*	% *	*n*	% *	*n*	% *	*n*	% *
Response after	no	7	41.2	12	70.6	3	37.5	7	87.5	4	44.4	5	55.6
melphalan	yes	10	58.8	5	29.4	5	62.5	1	12.5	5	55.6	4	44.4

* Column percentages.

## Data Availability

We understand the terms of the share upon reasonable request data policy. The data generated or analyzed during the study are available from the corresponding author upon request.

## Supplementary Materials

The following supporting information can be downloaded at: https://www.mdpi.com/article/10.3390/jcm12206542/s1, Table S1: Patient demographics and interventional characteristics.
