# Supplementary material for: The Use of Regional Anesthesia to Reduce Blood Loss in Isolated Limb Perfusion (ILP)—A Novel Approach
Source: J Clin Med. 2023 Oct 16;12(20):6542. doi: 10.3390/jcm12206542 (PMC10607281; doi:10.3390/jcm12206542)
Supplement: Supplementary file 1 [file jcm-12-06542-s001.zip › jcm-2626025-supplementary.pdf]

Supplementary Table S1: Patient demographics and interventional characteristics.

ID	sex	age	ILP arm/leg	surgical approach	regional anesthesia	PDK/SKP	TNF	Melphalan	extremity volume [l]	time of perfusion [min.]	leackage	RaM	substituted volume	surgical procedure [min.]	BLt [ml]	body hight [cm]	body weight [kg]	neoadjuvant CTX/RTX	preoperative Hb [g/dl]	preoperative Hc [%]	postoperative Hb [g/dl]	postoperative Hc [%]	postoperative transfusion
**Intervention Group**																							
RA01	m	80	arm	brachial	yes	SKP	1mg	30mg	4,54	94	<3%	no	4000ml	237	1400	174	74	no	11.7	36.5	6.5	19.5	1 RBC
RA02	m	48	arm	brachial	yes	SKP	1mg	40mg	4,08	92	< 1%	no	6000ml	216	700	180	92	CTX	12.0	35.2	10.1	31.0	
RA03	m	55	arm	brachial	yes	SKP	1mg	35mg	3,29	92	3,50%	no	7000ml	226	2000	181	93	CTX	15.4	43.2	8.1	23.0	
RA04	m	82	arm	brachial	yes	SKP	1mg	35mg	2,40	94	< 1%	no	3000ml	247	550	168	61	no	13.6	41.1	11.1	32.7	
RA05	m	64	arm	brachial	yes	SKP	1mg	40mg	2,78	95	5%	no	5000ml	245	1200	184	68	no	8.0	22.5	8.4	26.5	1 RBC
RA06	**m**	**15**	**leg***	**femoral**	yes	**PDK**	**1mg**	**60mg**	**7,06**	**69**	**< 1%**	**yes**	**5000ml**	132	**750**	**173**	**58**	**CTX**	**10.1**	**28.8**	**7.6**	**22.1**	**2 RBC**
RA07	**f**	**13**	**leg***	**femoral**	yes	**PDK**	**1mg**	**30mg**	**4,38**	**93**	**< 1%**	**no**	**3000ml**	227	**800**	**157**	**33**	**CTX**	**12.0**	**34.1**	**8.5**	**24.0**	**1 RBC**
RA08	**m**	**7**	**leg***	**femoral**	yes	**PDK**	**1mg**	**25mg**	**2,82**	**94**	**< 1%**	**no**	**3000ml**	197	**700**	**116**	**19**	**no**	**12.1**	**34.4**	**10.1**	**28.3**	**1 RBC**
RA09	m	82	leg	femoral	yes	PDK	2mg	80mg	10,91	97	2%	yes	5000ml	228	1400	168	87	no	12.8	37.6	8.0	23.8	
RA10	m	80	leg	iliacal	yes	PDK	2mg	120mg	12,32	92	2%	yes	5000ml	232	800	170	100	no	11.8	36.1	6.9	20.8	
RA11	f	67	leg	iliacal	yes	PDK	2mg	120mg	17,64	96	7%	no	6000ml	311	2500	165	119	no	15.0	45.2	11.4	32.9	
RA12	m	76	leg	femoral	yes	PDK	2mg	80mg	7,83	93	< 1%	no	5000ml	190	1500	170	70	no	9.1	30.2	6.8	20.5	2 RBC
RA13	m	49	leg	iliacal	yes	PDK	2mg	120mg	12,00	73	10%	no	8000ml	248	500	183	83	CTX	14.1	40.4	7.9	22.7	
RA14	m	65	leg	femoral	yes	PDK	2mg	70mg	10,30	93	< 4%	yes	7000ml	247	2500	170	94	no	15.8	43.4	7.8	21.7	
RA15	f	77	leg	femoral	yes	PDK	2mg	70mg	8,78	93	< 5%	no	3000ml	222	1200	162	71	CTX	10.6	30.8	6.4	18.5	1 RBC
RA16	f	81	leg	femoral	yes	PDK	2mg	70mg	9,39	93	<5%	yes	4000ml	193	1200	160	64	no	12.8	39.4	6.8	20.5	
RA17	f	83	leg	femoral	yes	PDK	2mg	60mg	9,60	90	< 1%	no	4000ml	219	1000	164	70	no	13.5	38.8	7.4	21.0	1 RBC

Control Group

AA01	m	78	arm	brachial	no	1mg	30mg	3,30	97	< 5%	yes	6000ml	266	2200	174	82	CTX/RTX	12.8	38.7	7.8	22.8	
AA02	m	75	arm	brachial	no	1mg	30mg	3,14	90	< 1%	no	6000ml	286	1300	170	77	CTX	10.6	30.6	7.5	20.9	2 RBC
AA03	m	55	arm	brachial	no	1mg	40mg	4,96	96	< 2%	yes	7000ml	235	1600	180	106	CTX	12.0	34.3	8.2	24.2	
AA04	m	56	arm	brachial	no	1mg	30mg	2,77	88	< 1%	yes	3000ml	201	1000	181	88	no	12.2	36.2	7.4	21.3	
AA05	m	17	arm	brachial	no	1mg	20mg	2,65	89	< 1%	yes	7000ml	231	2000	**180**	**80**	**CTX/RTX**	**10.5**	**30.6**	**8.4**	**23.6**	
AA06	m	83	arm	axillary	no	1mg	50mg	3,76	90	2%	no	5000ml	278	1100	180	80	no	13.9	40.2	9.9	27.2	
AA07	m	65	arm	brachial	no	1mg	30mg	3,73	94	< 1%	yes	4000ml	229	1400	196	92	CTX	11.2	33.1	6.1	17.6	
AA08	f	27	arm	brachial	no	1mg	20mg	1,89	89	< 5%	no	3000ml	240	600	164	49	no	13.9	39.0	9.0	24.8	
AA09	m	56	leg	femoral	no	2mg	100mg	12,84	93	8%	yes		237	2500	180	101	no	16.7	48.5	10.3	28.6	
AA10	f	30	leg	iliacal	no	2mg	120mg	13,81	88	< 1%	no	5000ml	243	1500	165	85	no	14.4	43.1	8.4	24.4	
AA11	m	36	leg	adductoral	no	2mg	100mg	14,37	90	< 1%	no	4000ml	260	1200	182	114	CTX	11.9	34.4	8.7	25.2	
AA12	f	81	leg	femoral	no	2mg	80mg	9,28	91	< 1%	no	5000ml	211	900	168	68	no	13.7	40.9	9.1	25.7	
AA13	m	25	leg	iliacal	no	2mg	100mg	10,62	92	< 1%	no	5500ml	240	1000	173	67	CTX	12.3	36.7	8.9	26.5	
AA14	m	51	leg	iliacal	no	2mg	100mg	11,35	86	< 5%	yes	7000ml	230	2500	180	86	CTX	9.5	28.8	7.5	21.2	3 RBC (+ 1 RBC prior to perfusion)
AA15	f	69	leg	femoral	no	2mg	80mg	7,85	94	< 3%	yes	5000ml	257	2400	172	68	no	13.0	37.8	8.5	24.6	2 RBC
AA16	f	23	leg	iliacal	no	2mg	80mg	7,94	94	< 0,5%	yes	5000ml	218	1500	175	59	RTX	12.7	38.4	6.4	18.2	
AA17	m	78	leg	femoral	no	2mg	80mg	10,95	93	< 1%	yes	5000ml	229	1000	181	79	no	11.3	35.0	7.5	21.2	

* ILP of the lower extremity with PDK in a child was assigned to the upper extremity intervention group because of comparable extremity volume and adapted dosage of melphalan and TNF-a.

ILP, isolated limb perfusion; PDK, peridural catheter; SKP, suprascalene block; RaM, response after melphalan; BLt, total blood loss; RBC, unit of red blood cells
